# Holt-Oram Syndrome in Adult Presenting with Heart Failure: A Rare Presentation

**DOI:** 10.1155/2014/130617

**Published:** 2014-03-23

**Authors:** Rupesh Kumar, Subhendu Sekhar Mahapatra, Monalisa Datta, Amanul Hoque, Swarnendu Datta, Soumyajit Ghosh, Santanu Datta, Subhankar Bhattacharjee

**Affiliations:** ^1^Department of Cardiothoracic and Vascular Surgery, Institute of Postgraduate Medical Education & Research, SSKM Hospital, AJC Bose Road, PO-Bhowanipore, Kolkata 700020, India; ^2^Department of Cardiothoracic and Vascular Anesthesiology, Institute of Postgraduate Medical Education & Research, SSKM Hospital, Kolkata 700020, India

## Abstract

Holt-Oram syndrome is a rare inherited disorder involving the hands, arms, and the heart. The defects involve carpal bones of the wrist and the thumb and the associated cardiac anomalies like atrial or ventricular septal defects. Congenital cardiac and upper-limb malformations frequently occur together and are classified as heart-hand syndromes. The most common amongst the heart-hand disorders is the Holt-Oram syndrome, which is characterized by septal defects of the heart and preaxial radial ray abnormalities. Its incidence is one in 100,000 live births. Approximately three out of four patients have some cardiac abnormality with common associations being either an atrial septal defect or ventricular septal defect. Herein, we report a rare sporadic case of Holt-Oram syndrome with atrial septal defect with symptoms of heart failure in a forty-five-year-old lady who underwent emergency cardiac surgery for the symptoms.

## 1. Introduction

Holt-Oram syndrome is a rare inherited disorder involving the hands, arms, and the heart. The defects involve carpal bones of the wrist and the thumb and the associated cardiac anomalies like atrial or ventricular septal defects. Congenital cardiac and upper-limb malformations frequently occur together and are classified as heart-hand syndromes. The most common amongst the heart-hand disorders is the Holt-Oram syndrome, which is characterized by septal defects of the heart and preaxial radial ray abnormalities. Its incidence is one in 100,000 live births. Cardiac defects include mainly atrial septal or ventricular septal defect, the electrocardiographic abnormalities ranges from asymptomatic conduction disturbance to variable degree of atrioventricular block. Rare but other cardiac associations include pulmonary stenosis, mitral valve prolapse, and arrhythmias in the form of atrioventricular blocks. More complex cardiac lesions such as tetralogy of Fallot, endocardial cushion defects, and total anomalous pulmonary venous return are also noted in these subjects. The cardiac disease may manifest as an emergency necessitating prompt intervention.

## 2. Case Report 

**Figure 1:**
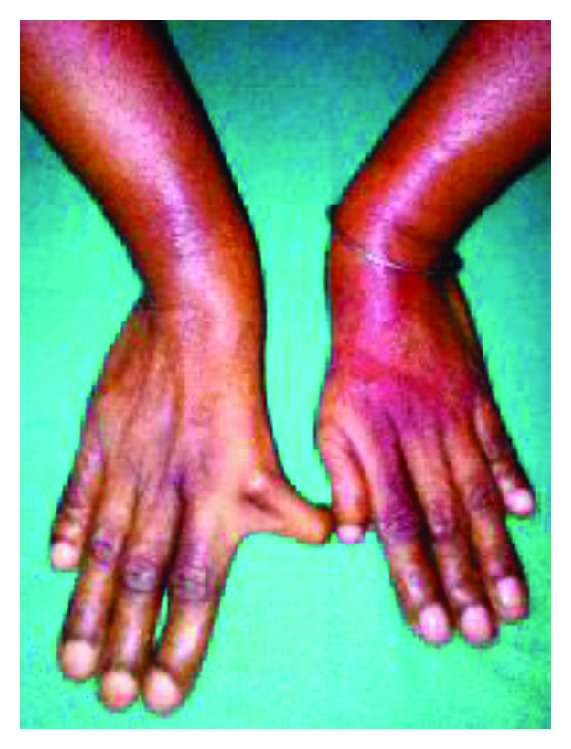
Rudiment thumb and little fingers with adductor deformity of both wrists.

A forty-year-old lady presented to our emergency department with symptoms of severe respiratory distress. She had a history of recurrent episodes of cough and cold and effort breathlessness since childhood. Physical examination revealed elevated jugular venous pressure, bilateral pedal edema, hepatomegaly, blood pressure of 104/66 mm of Hg, and respiratory rate of 38/min and systemic oxygen saturation was 85% at ambient room air. On musculoskeletal examination, she had an abnormally rudiment thumb and little fingers with adductor deformity of both wrists (Figure 1). No obvious deformities were observed in lower limbs or elsewhere. Cardiovascular examination revealed overactive left parasternal systolic lift, fixed splitting of the second heart sound, and a systolic murmur of grade IV/VI at left parasternal area.

**Figure 2:**
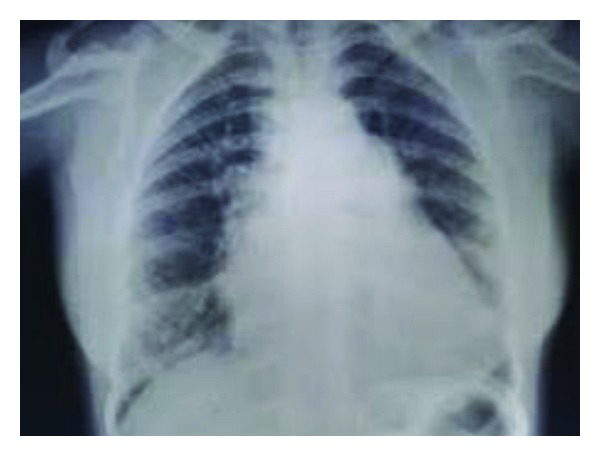
Cardiomegaly and increased pulmonary vascular markings.

**Figure 3:**
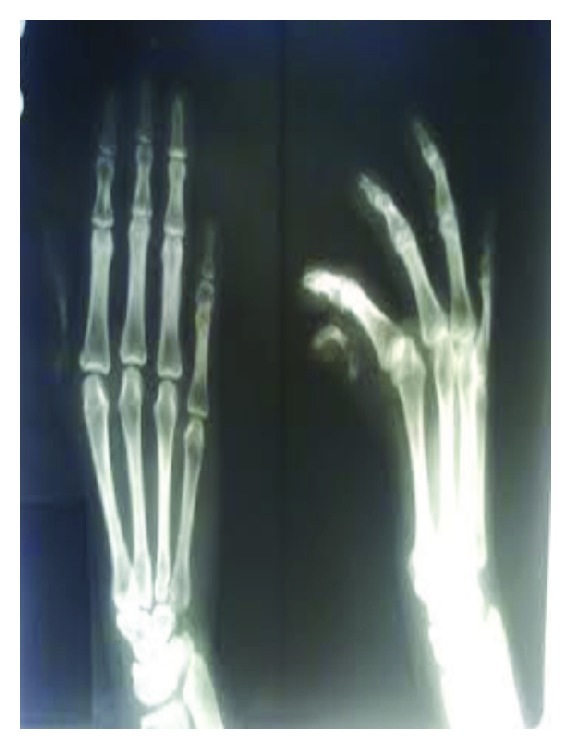
Absent first metacarpal bone of the left hand, underdeveloped distal phalanges of thumb and the little finger of both hands.

**Figure 4:**
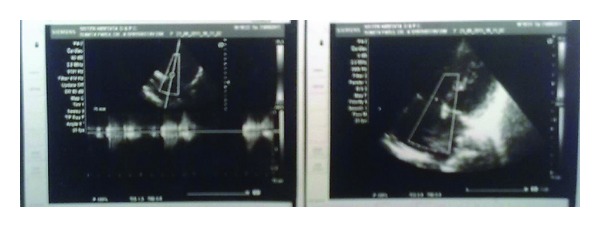
Echocardiographic view of ostium secundum atrial septal defect.

Electrocardiogram showed normal sinus rhythm with right ventricular hypertrophy with right axis deviation. Chest X-ray poster anterior view showed levocardia, cardiomegaly mainly of right atrium and the right ventricle, increased pulmonary vascular markings, and normal thoracic situs (Figure 2). Plain radiograph of both hands revealed absence of first metacarpal bone of the left hand, and underdeveloped distal phalanges of thumb and the little finger of both hands (Figure 3). Two-dimensional echocardiography showed ostium secundum atrial septal defect (Figure 4). Renal ultrasound and kidney function tests were normal. She was stabilized with medical management and underwent the complete intracardiac repair with pericardial patch closure of the septal defect under cardioplegic arrest on the second day of admission. On followup for the last two years, she is doing well and is asymptomatic regarding her cardiac symptoms without any medical therapy.

## 3. Discussion 

Holt-Oram syndrome (HOS) is also known as the atriodigital dysplasia syndrome [1]. It is an autosomal dominant disorder, caused by mutations on chromosome 12q24.1 that inactivate the TBX5 gene [2]. Holt and Oram first elaborated this familial syndrome in nine members of a family spanning four generations [3]. The prevalence of HOS is approximately one per 100,000 births with 85% cases occurring due to mutations. The clinical features of HOS are dysplasia of upper limb ranging from minor radiographic abnormalities to phocomelia and cardiac abnormalities. The skeletal deformities are mainly triphalangeal thumbs, shortness of ulna, shortness of the humerus, aplasia of the radius, dysmorphism of carpal bones, and phocomelia [4]. Skeletal abnormalities rarely involve the lower limbs. This is because the mutant gene interferes with the embryonic differentiation during the 4th and 5th weeks of pregnancy, when the lower limbs are not differentiated [5]. Several clinical and genetic studies noted that almost all cases of HOS had upper extremity involvement; the females had more severe anomalies. This feature matches with our case. Cardiac defects include mainly atrial septal or ventricular septal defect, electrocardiographic abnormalities ranges from asymptomatic conduction disturbance to variable degree of atrioventricular block. Rare but other cardiac associations include pulmonary stenosis, mitral valve prolapse, and arrhythmias in the form of atrioventricular blocks. More complex cardiac lesions such as tetralogy of Fallot, endocardial cushion defects, and total anomalous pulmonary venous return are also noted in subjects with HOS [6]. The degree of upper limb defects does not correlate with the severity cardiac examination. The skeletal deformities characterized by distinctive malformation of bones of the upper limbs and the abnormalities of heart are associated in different forms in different patients. HOS is also known as heart -hand syndromes which includes a constellation of defects and is characterized by deformities of the radial ray and congenital heart defects like thrombocytopenia, absent radius syndrome, Fanconi anaemia, Roberts syndrome, and thalidomide embryopathy. The unique feature that helps to differentiate these from HOS is that the radial aplasia is associated with hypoplasia/absence of the thumb without any haematological abnormalities and there is often a family history of heart and limb defects.

The associated congenital heart defects are the most important determining factors in morbidity and mortality in these patients. More than 85% of affected individuals have cardiac malformations particularly atrial septal or ventricular septal defects which may be simple or associated with other cardiac anomalies like pulmonary stenosis, mitral valve prolapse, and arrhythmias in the form of atrioventricular blocks.

In conclusion, we report a rare case of Holt-Oram syndrome with an associated atrial septal defect and upper extremity musculoskeletal deformity which is a very rare clinical entity where a clinician should have a very high index of suspicion of cardiovascular defect related emergency in any patients who have an associated congenital musculoskeletal defects. None of the studies have stressed on the HOS in adults presenting with symptoms of life threatening heart failure necessitating emergency cardiac surgery.
